# A tailored lectin microarray for rapid glycan profiling of therapeutic monoclonal antibodies

**DOI:** 10.1080/19420862.2024.2304268

**Published:** 2024-01-22

**Authors:** Shen Luo, Baolin Zhang

**Affiliations:** Office of Pharmaceutical Quality, Center for Drug Evaluation and Research, Food and Drug Administration, Silver Spring, MD, USA

**Keywords:** Bispecific antibody, glycan profile, glycosylation, lectin microarray, monoclonal antibody, product quality, therapeutic antibodies, FDA approval

## Abstract

Glycosylation plays a crucial role in determining the quality and efficacy of therapeutic antibodies. This necessitates a thorough analysis and monitoring process to ensure consistent product quality during manufacturing. In this study, we introduce a custom-designed lectin microarray featuring nine distinct lectins: rPhoSL, rOTH3, RCA120, rMan2, MAL_I, rPSL1a, PHAE, rMOA, and PHAL. These lectins have been specifically tailored to selectively bind to common N-glycan epitopes found in therapeutic IgG antibodies. By utilizing intact glycoprotein samples, our nine-lectin microarray provides a high-throughput platform for rapid glycan profiling, enabling comparative analysis of glycosylation patterns. Our results demonstrate the practical utility of this microarray in assessing glycosylation across various manufacturing batches or between biosimilar and innovator products. This capacity empowers informed decision-making in the development and production of therapeutic antibodies.

## Introduction

Many therapeutic antibodies, including bispecific and other monoclonal antibodies (mAbs), are glycoproteins produced in engineered cell lines. These glycoproteins contain glycans attached to a specific N-glycosylation site, often identified as Asn 297 within the crystallizable fragment (Fc) of IgG1 mAbs.^1^ These N-linked glycans play a crucial role in ensuring the proper folding, stability, and biological activity of glycoproteins, directly affecting the product’s safety and efficacy. For instance, in the case of IgG1 mAbs, Fc glycosylation can influence Fc effector functions, such as complement-dependent cytotoxicity (CDC) and antibody-dependent cellular cytotoxicity (ADCC).^2^ Additionally, recombinant mAbs may contain non-human glycoforms, such as N-glycolylneuraminic acid (NGNA) residues or Galα1-3 Gal disaccharide (α-Gal) units.^3,4^ These non-human glycoforms could potentially trigger an immune response in patients.

Glycosylation is a naturally occurring process characterized by its inherent heterogeneity, primarily due to its non-template driven biosynthesis machinery. Several factors, including the cellular expression systems, culture conditions, and purification methods, can influence this process.^5^ Consequently, the glycosylation patterns, encompassing the types and abundance of glycans, may vary between batches, leading to variations in product quality. As a result, glycosylation is considered a critical quality attribute (CQA) for specific therapeutic antibodies, including both novel modalities and biosimilars. It is of utmost importance to appropriately characterize and control glycosylation during the development of therapeutic antibodies to ensure consistent product quality and manufacturing.

There are various methods available for analyzing glycosylation, which allow for the assessment of glycosylation sites, glycan species and structures or epitopes, and their relative abundance. Many of these methods involve the combination of liquid chromatography with mass spectrometry (LC-MS) and fluorescence detection. Due to the intricate nature of glycosylation, it often requires a combination of analyses on intact glycoproteins, protein subunits, peptides, and released glycans.^6^ One approach to separate N-linked glycans from the protein backbone involves treating a glycoprotein sample with the enzyme peptide-N-glycosidase F (PNGase F).^7^ The resulting N-glycans are then labeled with a fluorophore dye and analyzed using LC-MS, typically utilizing hydrophilic-interaction chromatography (HILIC). However, this method has limitations. In particular, it is time-consuming, has low throughput, and may not fully digest or release all glycans, leaving some unreleased glycans unaccounted for.^8^ Other methods for glycosylation analysis include capillary electrophoresis-mass spectrometry (CE-MS), capillary electrophoresis-laser-induced fluorescence detection (LIF), high-performance anion-exchange chromatography with pulsed amperometry detection (HPAEC-PAD), and nuclear magnetic resonance (NMR) spectroscopy.^9^ Despite recent advancements in glycosylation analysis, there is ongoing interest in improving the performance of methods by utilizing intact glycoproteins with high throughput capabilities.

The lectin microarray is a unique platform used to analyze glycoproteins. It leverages the selective interactions between naturally-occurring or recombinant lectin proteins and specific glycan epitopes.^10–14^ This method involves immobilizing lectins to a solid surface, labeling the glycoproteins in the sample, and detecting the fluorescently labeled glycoproteins with a specialized evanescent-field activated fluorescence detection system.^15^ Notably, this approach eliminates the need for washing steps and allows for direct observation in a liquid state. In our previous evaluation, we assessed a commercial lectin microarray comprising 45 lectins. This array demonstrated its capacity to recognize a wide range of glycan epitopes. However, it is important to note that some of these lectins exhibited limited selectivity for certain glycans found in therapeutic mAbs. This lack of selectivity led to false-positive or inconclusive binding signals.^16^

**Figure 1.**
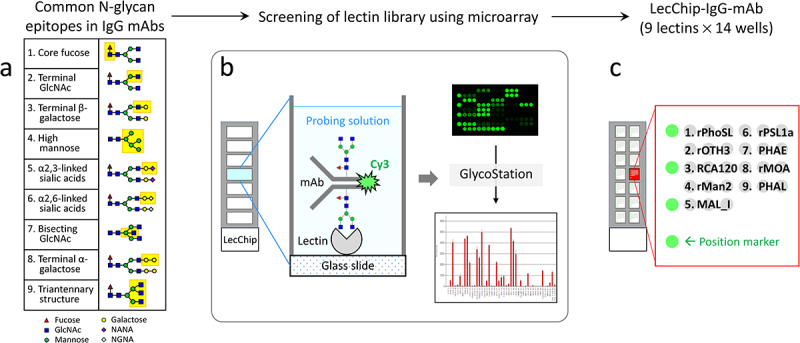
Workflow for the development of lectin chips for IgG mAbs. The left panel illustrates the nine glycan epitopes found in therapeutic IgG mAbs, which served as a reference for identifying lectins as binding partners. A total of 74 lectins, including 45 naturally occurring lectins and 29 recombinant lectins, were printed onto glass chips. These lectin chips were then exposed to Cy3-labeled glycoprotein samples with known glycan profiles, such as IgG1 mAbs and other therapeutic glycoproteins. The resulting binding signals were compared to the known selectivity of individual lectins, enabling the identification of nine distinct lectins that exhibited desirable selectivity toward specific glycan epitopes. Subsequently, these nine lectins were used to fabricate the lectin microarray chips, referred to as the LecChip-IgG-mAb.

To address this limitation, our present study focuses on developing a new type of lectin microarray chips specifically designed for therapeutic IgG mAbs. In this pursuit, we have identified nine universal glycan epitopes that are common to all therapeutic mAbs produced through mammalian cell expression systems, as approved by the US Food and Drug Administration (FDA).^17^ These epitopes encompass core fucose, terminal N-acetylglucosamine, terminal β-galactose, high mannose, terminal α2,3-linked N-acetylneuraminic acid, terminal α2,6-linked N-glycolylneuraminic acid, bisecting N-acetylglucosamine, α-galactose, and triantennary N-glycan (Figure 1a). In this context, the term ‘epitope’ specifically refers to the distinct carbohydrate structures recognized by lectins. Like antibodies identifying antigen epitopes, lectins, as carbohydrate-binding proteins, identify specific carbohydrate structures on glycoproteins. By using ‘epitope’ in this context, we clarify how lectins recognize particular carbohydrate patterns on glycoproteins. Subsequently, we have used these epitopes as a standard to create and validate a specialized lectin microarray tailored for the precise characterization of IgG mAbs produced through various expression systems.

## Results

### Lectin library screening

To pinpoint lectins capable of specifically recognizing the nine distinct glycan epitopes present in therapeutic mAbs, we conducted a rigorous screening process. This involved use of a commercial lectin library comprising 74 lectins. The library encompassed 45 naturally occurring lectins and 29 recombinant lectin proteins, each exhibiting distinct binding selectivity toward various glycan epitopes (Supplemental Tables S1 and S2). These lectins were supplied by GlycoTechnica (Japan) and were affixed to two sets of glass chips: one set contained the 45 natural lectins while the other set immobilized a combination of 29 recombinant lectins and 16 of the 45 natural lectins. Each chip featured triplicate samples of 45 lectins and 7 wells for testing 7 samples (Figure 1b).

The lectin chips were incubated with Cy3-labeled glycoprotein samples. Subsequently, fluorescence intensities were measured, as illustrated in Figure 1b, following a previously established protocol.^16^ This approach facilitated the efficient acquisition of large datasets pertaining to the interactions between individual lectins and the tested glycoprotein samples. These samples included a variety of controls and distinct glycan profiles. Specifically, we included a positive control, NISTmAb, which is a fully glycosylated IgG1 mAb, as well as two negative controls: filgrastim (a non-glycosylated therapeutic protein produced in *E. coli*) and atezolizumab (a non-glycosylated therapeutic IgG1 mAb).

We analyzed the binding signals at each lectin spot in relation to the known N-glycan epitopes of the glycoproteins under examination, taking into account three factors: 1) Correlation between the detected binding signals and the established selectivity of a lectin to a specific glycan epitope found within the test samples, 2) Exclusion of 17 lectins that displayed nonspecific binding signals to non-glycosylated protein samples, particularly to the non-glycosylated IgG1 atezolizumab (Supplemental Figure S1, Table S3), and 3). Lectins with a known affinity for O-glycans were also excluded, as O-glycans are not detectable in therapeutic IgG mAbs (refer to Supplemental Tables S1 and S2). Based on these criteria, we identified nine distinct lectins that selectively recognize nine corresponding glycan epitopes present in therapeutic IgG mAbs. These nine lectins are as follows: rPhoSL,^18^ rOTH3,^19^ RCA120, rMan2,^13^ MAL_I, rPSL1a,^20^ PHAE, rMOA,^21^ and PHAL (Table 1). To further advance our research, we used these nine lectins to create a new generation of lectin microarray, which we have designated as LecChip-IgG-mAb. Each lectin, along with position marker (Cy3-labeled BSA), was printed in triplicates (Figure 1c).

**Table 1.** Selected lectins (*n* = 9) for capturing the common glycan epitopes of therapeutic IgG mAbs.

#	Lectin (origin)	Reported epitope selectivity relevant to the benchmark N-glycan epitope*	Targeting N-glycan epitope on IgG mAb
1	Recombinant PhoSL or rPhoSL (*Pholiota squarrosa*)	α(1,6)fucosylated N-glycans^18^	Core fucose (Fuc)
2	rOTH3 (*Ulva limnetica*)	Unknown (see Supplemental Table S2)^19^	Terminal N-acetylglucosamine (GlcNAc)
3	RCA120 (*Ricinus communis*)	Galβ(1,4)GlcNAc (up with increasing the number of terminal β-Gal), Galβ(1,3)Gal (weak), no affinity for agalactosylated N-type and α-galactose	Terminal β-galactose (β-Gal)
4	rMan2 (*Kappaphycus alvarezii*)	High Mannose (High Man)^13^	High mannose (High Man)
5	MAL_I (*Maackia amurensis*)	Siaα(2,3)Galβ(1,4)GlcNAc	Terminal α2,3-linked sialic acids; primarily N-acetylneuraminic acid (NANA) in CHO-produced mAbs
6	rPSL1a	Siaα(2,6)Galβ(1,4)GlcNAc^20^	Terminal α2,6-linked sialic acids, primarily N-glycolylneuraminic acid (NGNA) in murine cell produced mAbs
7	PHAE (*Phaseolus vulgaris*)	Bi-antennary complex-type N-glycan with outer β-Gal and bisecting GlcNAc (up with increasing the number of terminal β-galactose), no affinity for fully sialylated N-type	Bisecting GlcNAc
8	rMOA (*Marasmius oreades*)	α-Gal (Galα(1,3)Galβ(1,4)GlcNAc),^21^ no affinity for β-galactose	α-galactose (α-Gal)
9	PHAL (*Phaseolus vulgaris*)	Tri/tetra-antennary complex-type N-glycan	Triantennary N-glycan

*For naturally occurring lectins, refer to Lectin Frontier DataBase (LfDB)(https://acgg.asia/lfdb2/).

### Evaluation of LecChip-IgG-mAb performance using model samples

We evaluated the performance of the LecChip-IgG-mAb, which incorporates nine lectins, by conducting tests on a panel of 14 protein samples, each with distinct glycan profiles (refer to Table 2).

**Table 2.** Therapeutic protein samples tested using the LecChip-IgG-mAb microarray.

No.	Nonproprietary name	Proprietary name	Featured N-glycan epitope	Protein class	Expression cell lines*^1^
1	Filgrastim	Neupogen	Nonglycosylated protein, general negative control	Cytokine	*E. coli*
2	Atezolizumab	Tecentriq	Nonglycosylated IgG1, IgG1 negative control	IgG1 mAb	CHO^22^
3	NISTmAb	Not applicable	Well characterized N-glycosylated IgG1 mAb, a reference material widely used for analytical method development	IgG1 mAb	NS0
4	Trastuzumab	Herceptin	Typical IgG1 produced in CHO	IgG1 mAb	CHO
5	Cetuximab	Erbitux	Unique IgG1 with 2 N-glycosylation sites on heavy chain in both the Fc and the Fab domain, contains NGNA and α-Gal glycans in its Fab region	IgG1 mAb	Sp2/0^23^
6	Benralizumab	Fasenra	Afucosylated, terminal GlcNAc	IgG1 mAb	CHO
7	Siltuximab	Sylvant	High mannose	IgG1 mAb	CHO
8	Ramucirumab	Cyramza	α2,6-sialylation with NGNA, α-Gal	IgG1 mAb	NS0
9	Obinutuzumab	Gazyva	Bisecting GlcNAc, appears to contain low abundance of core fucose	IgG1 mAb	CHO
10	Darbepoetin alfa	Aranesp	Tri/tetra-antennary N-glycans, α2,3-sialylation with NANA	Cytokine	CHO
11	Infliximab	Remicade	Innovator product	IgG1 mAb	Sp2/0
12	Infliximab-dyyb	Inflectra	Biosimilar produced in the same cell line	IgG1 mAb	Sp2/0
13	Infliximab-abda	Renflexis	Biosimilar produced in a different cell line	IgG1 mAb	CHO^24^
14	Infliximab-axxq	Avsola	Biosimilar produced in a different cell line	IgG1 mAb	CHO
15	Etanercept	Enbrel	Other type of IgG mAb with 2 *N*- and multiple O-glycosylation sites on the fusion-protein domain in addition to IgG1-Fc N-glycosylation, high level of β-Gal and high mannose	IgG1-Fc fusion protein	CHO
16	Ado-trastuzumab emtansine	Kadcyla	Other type of IgG mAb with typical N-glycan profile	IgG1 ADC	CHO
17	Panitumumab	Vectibix	Other type of IgG mAb with high mannose	IgG2 mAb	CHO
18	Pembrolizumab	Keytruda	Other type of IgG mAb with typical N-glycan profile	IgG4 mAb	CHO

* *E. coli, Escherichia coli; CHO, Chinese Hamster Ovary; NS0 & Sp2/0 are two murine cell lines. 1. https://www.fda.gov/science-research/bioinformatics-tools/fdalabel-full-text-search-drug-product-labeling*.

This panel comprised 10 commercially available therapeutic IgG1 mAbs (trastuzumab, cetuximab, benralizumab, siltuximab, ramucirumab, obinutuzumab, infliximab, infliximab-dyyb, infliximab-abda, infliximab-axxq), one recombinant glycoprotein (darbepoetin alfa), the NISTmAb reference material, and two non-glycosylated therapeutic proteins (filgrastim, IgG1 atezolizumab). These samples were chosen as model samples for glycan analysis due to their diverse expression systems (CHO, Sp2/0, NS0) and well-documented glycan profiles (Table 2).

**Figure 2.**
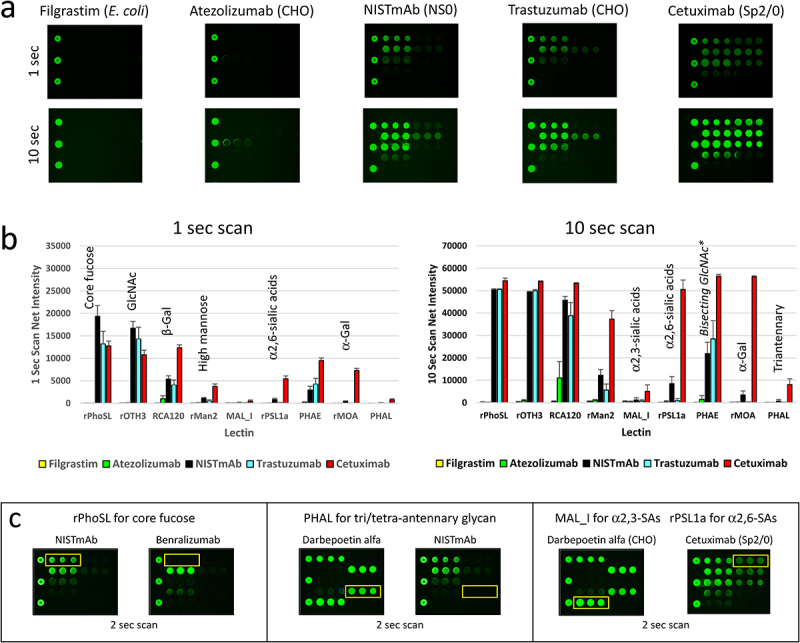
Qualification of LecChip-IgG-mAb using model samples with known glycan profiles. Two non-glycosylated therapeutic proteins (filgrastim and IgG1 atezolizumab), along with three N-glycosylated IgG1 mAbs produced by different cell lines (NISTmAb, trastuzumab, and cetuximab) were subjected to testing using LecChip-IgG-mAb (see details in the materials and methods section). Representative raw images acquired at 1-second scan and 10-second scan are shown (a). The LecChip binding signals (b) were used to determine the relative abundance of individual N-glycan epitopes based on the known selectivity of each lectin (table 1). The error bars represent standard deviation (*n* = 3) derived from three independent experiments.*PHAE signal should only be used to evaluate samples containing predominantly bisecting glycans (see glycoengineering section). Additionally, a saturated signal was detected at approximately 50,000 net fluorescence intensity, exceeding the lectin chips’ dynamic range. For a more detailed analysis of specific N-glycan epitopes (core fucose, triantennary N-glycan, and sialic acids (SAs)) among glycoprotein samples, three paired samples were applied onto the LecChip-IgG-mAb and scanned after a 2 second exposure (c). The yellow line box on each image indicates the location of triplicate spots for each lectin specified above the image.

To validate the specificity of LecChip, we included two negative controls, filgrastim and atezolizumab, both of which are non-glycosylated therapeutic proteins. As expected, we observed no binding signals across the entire chip coated with the nine lectins (Figure 2a). In contrast, all the glycoprotein samples exhibited binding signals of varying intensities at each lectin spot. For instance, NISTmAb and trastuzumab displayed strong binding to rPhoSL and rOTH3, which became visible after 1 second of chip scanning. This confirmed the presence of core fucose (e.g., G0F, G1F, G2F) and terminal GlcNAc (e.g., G0F). Longer exposure (10 seconds) revealed additional binding at RCA120 and rMan2, suggesting recognition of terminal β-Gal (e.g., G2F) and high mannose (e.g., M5), respectively.

**Figure 3.**
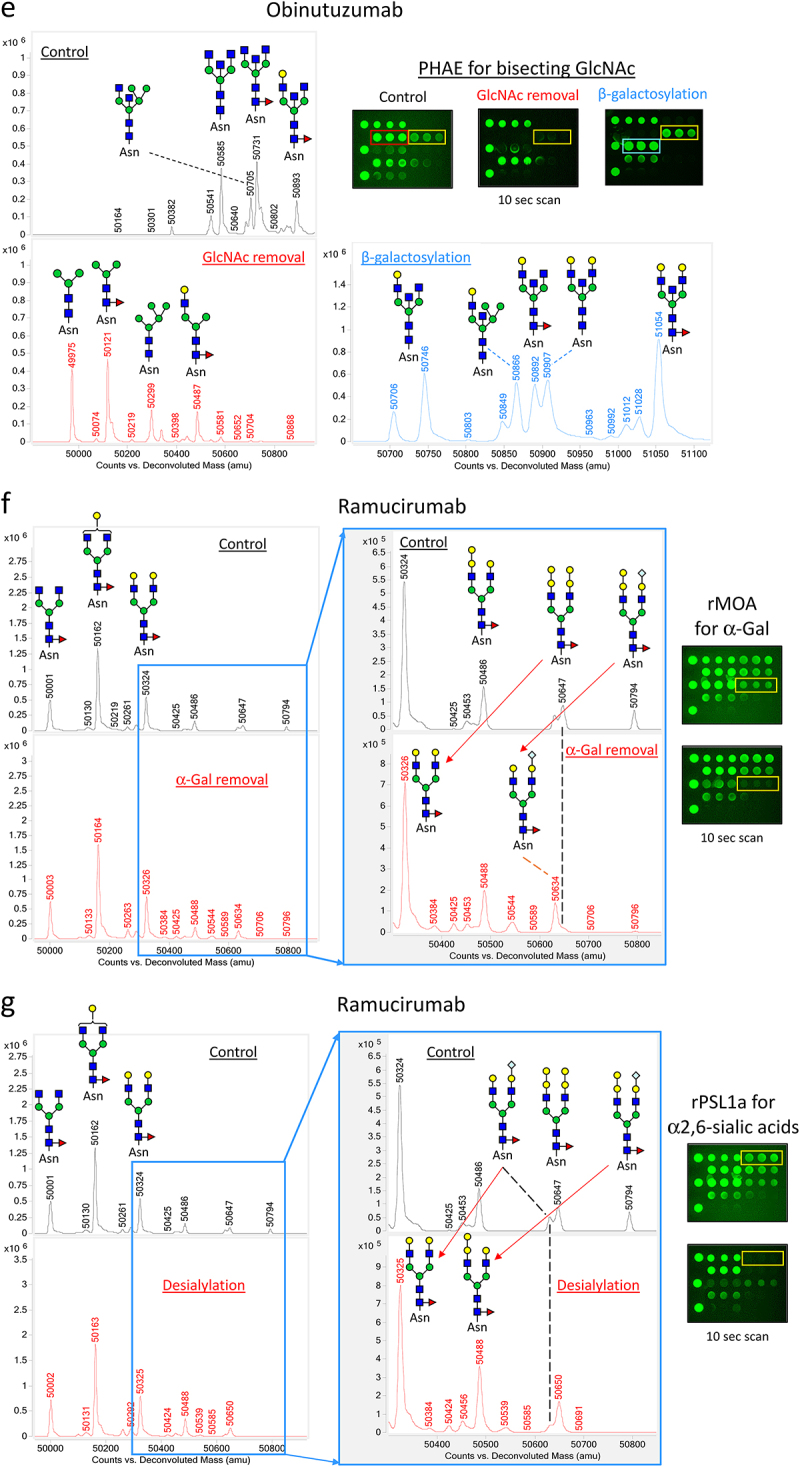
(Continued).

Cetuximab, which is known to possess two N-glycosylation sites on Fc and Fab regions of the IgG1 mAb,^23,25^ displayed distinct lectin-binding patterns. Specifically, it exhibited binding to rPSL1a and rMOA (as depicted in Figure 2a,b). These two recombinant lectins are known to recognize α2,6-linked sialic acids^20^ and α-Gal,^21^ respectively. This aligns with the reported epitopes associated with the top three N-glycans on the Fab region of the Sp2/0-produced cetuximab, namely G2F + 2αGal, G2F+αGal+NGNA, and G2F+NGNA.^3,23,26^ Additionally, rPhoSL demonstrated strong binding to fucosylated NISTmAb (as shown in Figure 2c, left panel, and Figure 3a, top mass spectrum), but not to the afucosylated benralizumab (Figure 2c, left panel, and Figure 3b top mass spectrum). This result confirms the selectivity of rPhoSL for core fucose.
**Figure 3.**
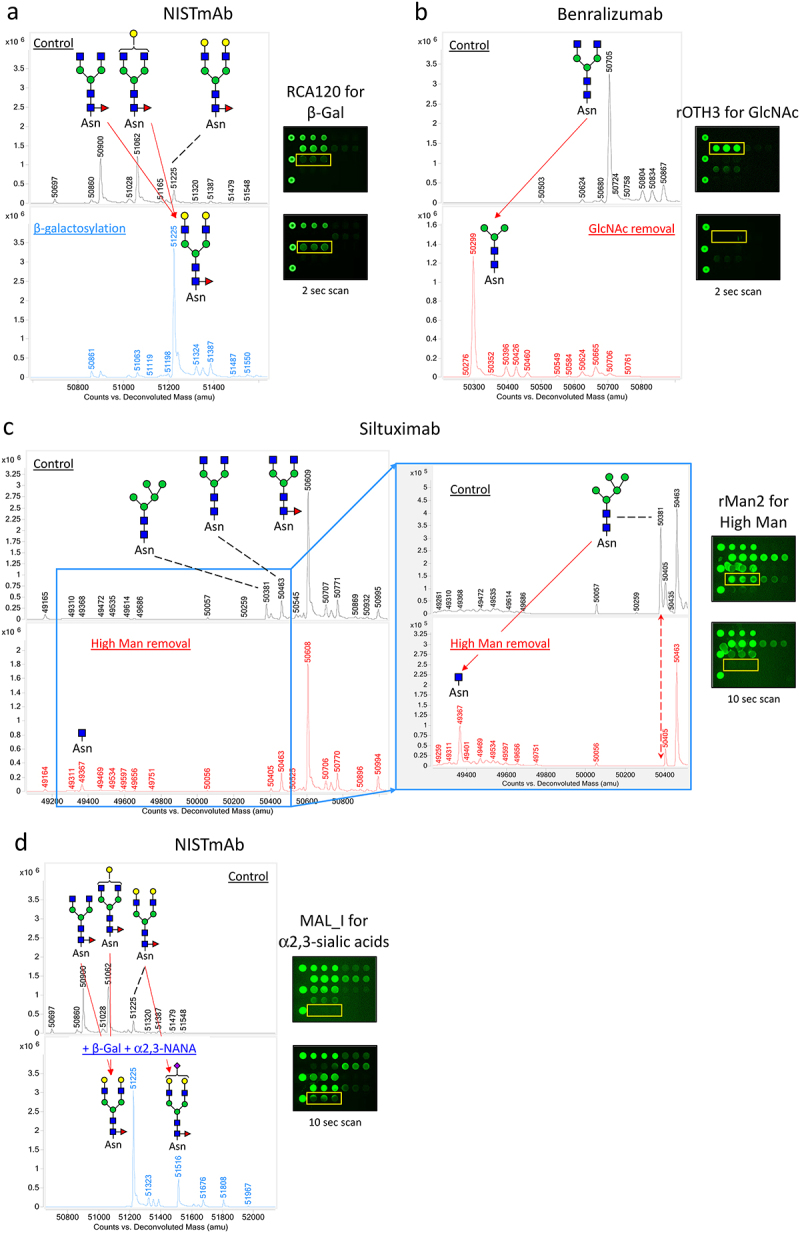
Qualification of LecChip-IgG-mAb through targeted glycoengineering of therapeutic IgG1 mAbs. The terminal N-glycan epitopes of the IgG1 mAbs shown in panels a to g were subjected to in vitro glycoengineering, as described in the materials and methods. The modified glycoforms were then confirmed through mass spectrometry, utilizing reduced intact protein samples (a-g, left panels). In parallel, a separate set of samples was analyzed using lectin microarray (a-g, right panels), which revealed consistent glycan profiles across all tested samples.

On the other hand, PHAL exhibited strong binding to darbepoetin alfa (as seen in Figure 2c, middle panel), which predominantly contains tri/tetra-antennary N-glycans.^27,28^ In contrast, it showed very weak binding to NISTmAb (Figure 2c, middle panel), which contains fewer tri-antennary N-glycans.^7^ This data underscores the specificity of PHAL for triantennary N-glycans on IgG1 mAb.

Moreover, it is established that CHO-produced darbepoetin alfa exclusively contains α2,3-linked terminal NANA at a high level,^27,28^ whereas Sp2/0-produced cetuximab mainly carries α2,6-linked terminal NGNA at a relatively lower level.^3,26,29^ This observation aligns with the finding that darbepoetin alfa exhibited strong binding to MAL_I but not to rPSL1a (Figure 2c right panel). Conversely, cetuximab predominantly bound to rPSL1a (Figure 2c right panel). The data confirm that MAL_I is specific for α2,3-linked sialic acids, while rPSL1a targets α2,6-linked sialic acids. In mAbs produced by CHO cells, the primary sialic acid form is NANA, whereas in mAbs from murine cells, it is mainly NGNA. As of our current knowledge, there are no identified pairs of lectins capable of distinguishing between NANA and NGNA. So, if an IgG mAb carries glycans with both α2,3-linked NGNA and α2,6-linked NANA, our interpretation of MAL_I and rPSL1a signals would accurately reflect the linkages, but not differentiate between the specific sialic acid types.

### LecChip-IgG-mAb specificity confirmed by glycoengineering of IgG1 mAbs

To further validate the specificity of LecChip, we conducted in vitro glycoengineering reactions to modify the terminal glycans of IgG1 mAbs. By employing specific glycosidases to remove glycan epitopes and glycosyltransferases to add glycan epitopes, we created a panel of glycoengineered samples with the desired terminal glycan epitopes (refer to Materials and Methods for detailed procedures). The resulting glycoforms were confirmed using intact protein mass spectrometry under reducing conditions (Figure 3). Subsequently, these protein samples underwent analysis using LecChip assays, which generated distinct lectin-binding profiles corresponding to the terminal glycan variations (Figure 3). Our data provide key evidence regarding specific lectin-glycan interactions, as follows. First, when NISTmAb underwent galactosylation, it converted glycans G0F and G1F to G2F, significantly enhancing its interaction with RCA120 (Figure 3a). This outcome confirms the selectivity of RCA120 for terminal β-galactose (β-Gal). Second, rOTH3 exhibited strong binding to benralizumab, rich in G0 glycan, but showed no affinity for its de-GlcNAc form lacking all the terminal GlcNAc from G0 (Figure 3b). This underscores rOTH3’s specificity for terminal GlcNAc. The rMan2 binding signal also exhibited a slight reduction after GlcNAc removal (Figure 3b), requiring further quantitative investigation to confirm the observation. Third, after the removal of terminal high mannose using endoglycosidase Endo H, siltuximab lost its binding affinity to rMan2 (Figure 3c), verifying rMan2’s selectivity for high mannose epitopes. Fourth, although MAL_I initially exhibited weak binding to NISTmAb, its affinity notably increased upon NISTmAb modification through α2,3-sialylation, resulting in a higher level of G2F+NANA glycan structure (Figure 3d). Therefore, MAL_I appeared to specifically bind to α2,3-linked terminal sialic acids on IgG1 mAbs, primarily the NANA structure in CHO-produced mAbs. Fifth, PHAE (yellow line box) predominantly binds to obinutuzumab with bisecting glycans (Figure 3e). This binding is reduced when the bisecting GlcNAc is removed. In contrast, β-galactosylation of the terminal GlcNAc significantly enhances RCA120 signals (blue line box), thereby increasing PHAE binding while decreasing rOTH3 signals (red line box). These results collectively emphasize the distinct selectivity of PHAE and rOTH3 for bisecting GlcNAc and terminal GlcNAc, respectively. It is worth noting that, due to reported interactions between PHAE and non-bisecting but abundant glycans in other mAb samples (e.g., G0F, G1F, G2F; Supplemental Figure S2), the PHAE signal should be specifically used to evaluate samples containing predominantly bisecting glycans. Sixth, rMOA’s binding to ramucirumab was eliminated after removing the terminal α-Gal from G2F + 2αGal and G2F+αGal+NGNA (Figure 3f), indicating rMOA’s specificity for the terminal α-Gal. It is noteworthy that rMOA exhibited a strong interaction with cetuximab (Figure 2b), a unique IgG1 with two N-glycosylation sites on the heavy chain present in both the Fc and the Fab domains. The α-Gal was reported to be present predominantly on the Fab glycans.^23^ Finally, rPSL1a selectively bound to ramucirumab containing a terminal NGNA on G2F+NGNA and G2F+αGal+NGNA, but not to the desialylated sample (Figure 3g). Additionally, lectin MAL_I did not bind to NS0-produced ramucirumab, indicating an α2,6-linkage between NGNA and G2F, consistent with reported glycan structures on IgG1 mAbs produced in murine cells.^7^ However, considering the reported specificity of rPSL1a for α2,6-linked NANA,^20^ it is evident that rPSL1a is specific for α2,6-linked sialic acids, but cannot distinguish NGNA from NANA. Consequently, the lectin rPSL1a is validated for detecting α2,6-linked sialic acids in IgG1 mAbs, which are primarily NGNA in murine cell-produced mAbs.

### Comparative glycan profiling of an innovator IgG1 mAb and its biosimilars

**Figure 4.**
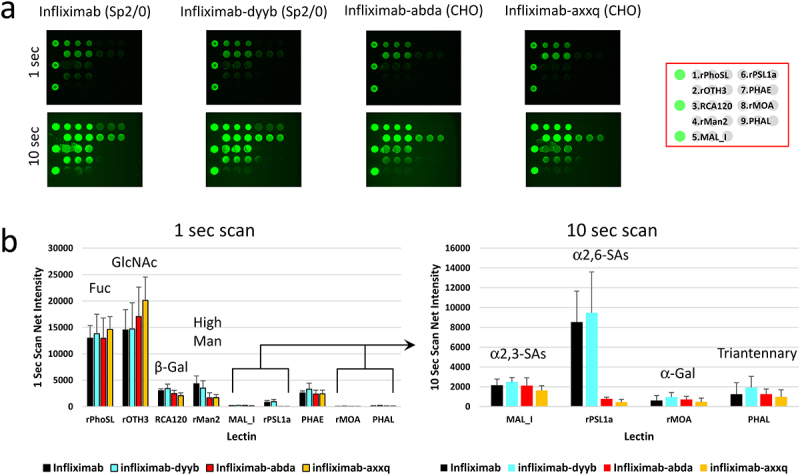
Comparative glycan profiling of infliximab and its biosimilars produced in two different expression systems. Infliximab and its biosimilars, infliximab-dyyb infliximab-abda, and infliximab-axxq, were subjected to analysis using the LecChip-IgG-mAb. Shown are the microarray images (a) and glycan profiles (b) derived from 1-second and 10-second exposures. The error bars represent standard deviation (*n* = 3) from three independent experiments.

To further assess the utility of the LecChip-IgG-mAb, we conducted comparative analyses involving an innovator IgG1 mAb and three of its biosimilar counterparts: infliximab, infliximab-dyyb, infliximab-abda, and infliximab-axxq (Figure 4). These samples exhibited similar levels of the major glycan epitopes, such as core fucose, terminal GlcNAc, and terminal β-galactose. However, notable differences emerged in the levels of high mannose and other minor glycan epitopes. Infliximabs produced in Sp2/0 cells had slightly higher levels of high mannose compared to infliximabs produced in CHO cells. Additionally, Sp2/0-produced infliximab exhibited lower levels of sialylated glycans, particularly the α2,6-linked NGNA species, in contrast to the almost negligible NGNA found in CHO-produced infliximabs. These findings align with data previously reported in a separate study.^24^ Additionally, the lectin microarray analysis of three batches of the infliximab drug product showed comparable glycan profiles, indicating manufacturing consistency (Supplemental Figure S3).

### Examining glycan profiles of other IgG mAbs

**Figure 5.**
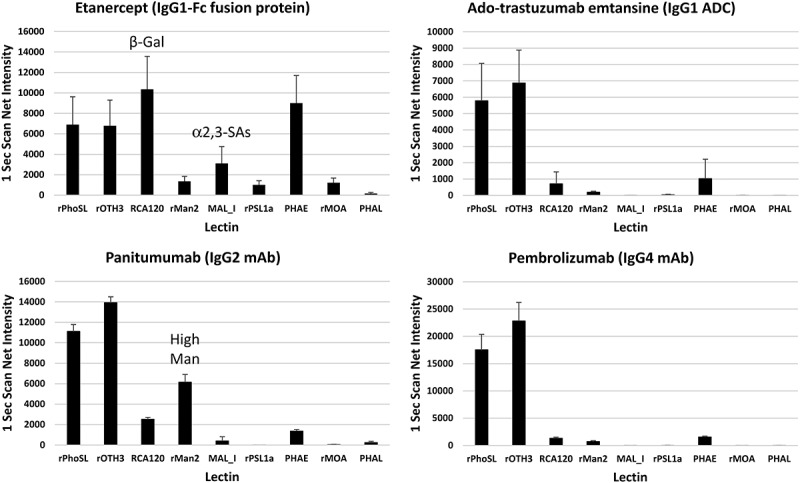
Applications of LecChip-IgG-mAb in glycan profiling of various mAb types. The IgG1-Fc fusion protein etanercept, IgG1 ADC ado-trastuzumab emtansine, IgG2 mAb panitumumab, and IgG4 mAb pembrolizumab, all produced in CHO cells, were analyzed using the LecChip-IgG-mAb. Shown are the glycan profiles obtained from 1-second exposures. The error bars represent standard deviation (*n* = 3) from three independent experiments.

The nine N-glycan epitopes commonly found in prevalent glycans among FDA-approved mAb products^17,30^ were explored using the LecChip-IgG-mAb microarray on different types of IgG mAbs (Table 2). The analysis included an Fc-fusion protein, an antibody-drug conjugate (ADC), and two non-IgG1 mAbs (Figure 5). Etanercept, an IgG1-Fc fusion protein, has unique N-glycan due to its reported two *N*- and multiple O-glycosylation sites on its fusion-protein domain, in addition to Fc N-glycosylation. This resulted in distinctive profile with high levels of G2F and NANA.^31^ The strong RCA120 and MAL_I signals on the microarray align with this profile, suggesting minimal interference, if any, from O-glycans on the nine-lectin microarray. The IgG2 mAb panitumumab was reported to have an elevated level of high mannose,^32^ which supports the observed strong rMan2 signal. Conversely, the IgG1 ADC ado-trastuzumab emtansine and IgG4 mAb pembrolizumab exhibited typical IgG1 glycan profiles, consistent with those found in trastuzumab (Figure 2b) or reported in pembrolizumab.^33^ Furthermore, antibody-drug conjugation appears to have no discernible impact on the lectin microarray.

## Discussion

Glycosylation plays a crucial role as a CQA in various therapeutic mAbs, including bispecific antibodies. It requires thorough analysis and precise control at every stage of product development and throughout the product’s lifecycle. Currently, industry practices use an integrated approach that emphasizes cell line engineering, process optimization, and analytical improvements. Consequently, it is imperative to monitor glycan variations for tasks such as clone selection, bioprocess parameter design, and the ultimate characterization of glycoproteins.

**Figure 6.**
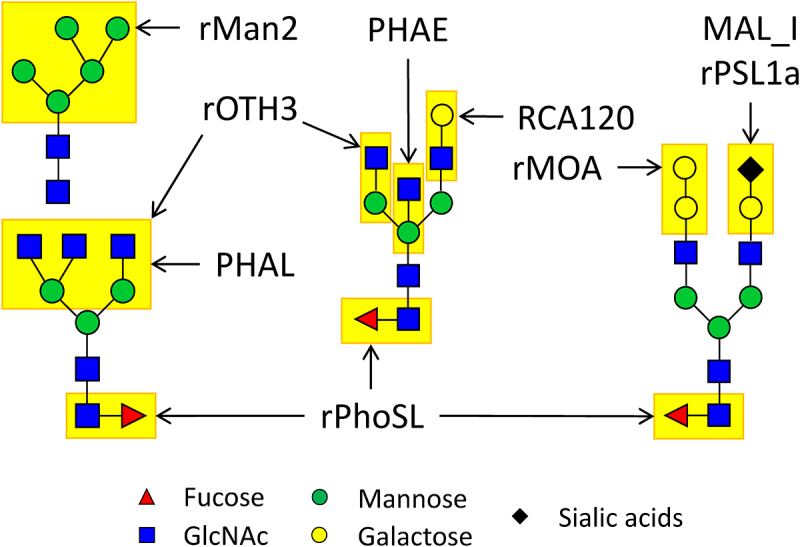
Schematic view of N-glycan epitopes identified on IgG mAbs, anticipated to engage with the nine lectins on the LecChip-IgG-mAb. Notably, lectin MAL_I binds to α-2,3 linkages, while rPsl1a recognizes α-2.6 linkages, primarily associated with NANA and NGNA present on glycans produced within CHO and murine cells.

To fulfill this need, we have introduced a novel nine-lectin microarray chip designed with nine distinct lectins, each capable of identifying a specific glycan epitope (Table 1, Figure 6) commonly present in therapeutic IgG mAbs.^17^ This high-throughput platform enables comparative glycan profiling of intact mAbs, eliminating the necessity of glycan release. Our panel testing of therapeutic IgG mAbs, encompassing glycoengineered versions and biosimilars, effectively showcased the utility of this microarray. It successfully profiled the glycans of various IgG mAbs, including IgG1, IgG2, IgG4, ADC, and Fc-fusion proteins. Our findings aligned with previous reports, highlighting core fucose on predominant G0F and G1F glycans in NISTmAb (Figures 2c and 3a),^34^ terminal GlcNAc on the main G0 glycan in benralizumab (Figure 3b),^35^ terminal β-galactose, high mannose, α2,6-linked sialic acid, and α-galactose in cetuximab (Figures 2a, 2b),^23,25,26^ and elevated levels of tri/tetra-antennary and α2,3-linked sialic acids in CHO-produced protein darbepoetin alfa (Figure 2c).^27,28^ Additionally, our nine-lectin microarray exhibited remarkable sensitivity to terminal glycan alterations, exemplified by targeted glycoengineering of IgG1 mAbs, encompassing galactosylation, α2,3-sialylation, and removal of terminal monosaccharide such as GlcNAc, high mannose, bisecting GlcNAc, α-galactose, and α2,6-sialic acids (Figure 3). Crucially, these nine epitopes are commonly found in FDA-approved mAb products, making our nine-lectin microarray a versatile tool for comprehensive glycan profiling across various therapeutic IgG mAbs and Fc-fusion proteins.^17,30^

One significant advantage of the microarray is its capacity to analyze intact glycoprotein samples, eliminating the variability linked to conventional methods that require glycans to be released from the protein structure. This procedure is straightforward, offers high throughput capacity, and allows for comparative testing of numerous samples within a short timeframe. However, it should be noted that our nine-lectin microarray, in its prototype phase, generates qualitative or semi-quantitative results. While it consistently identified glycan patterns, we noticed signal intensity variations for specific glycans over time. These differences might arise from chip-to-chip variations, the desalting process, or potential interference caused by Cy3 labeling. Despite these variations, our nine-lectin microarray remains a valuable tool for glycan profiling. With improvements in chip quality and assay protocols, it can be customized for various applications, enabling rapid glycan profiling.

Commercially marketed mAbs are predominantly produced using three expression systems, with CHO cells being the most prevalent, along with NS0 and Sp2/0 cells.^17^ These cell lines boast well-established infrastructure and a proven regulatory track record concerning viral safety and process controls, rendering them the preferred choice for therapeutic protein production within the industry. When these cell lines serve as production systems, variations in glycosylation primarily manifest at the terminal glycan structures. In this context, our newly developed nine-lectin microarray has showcased its unique capability to reliably detect terminal glycan profiles. Hence, it holds substantial promise for adoption by pharmaceutical companies during the development of IgG mAbs to assess the glycan epitopes of batch-to-batch variations or biosimilars in comparison to innovator products.

## Materials and methods

### Pharmaceutical protein products and critical reagents

Pharmaceutical-grade mAbs and protein products were purchased from commercial sources through a contract pharmacy service. NIST Monoclonal Antibody Reference Material 8671 (NISTmAb) and Cy3 mono-reactive dye were obtained from Sigma-Aldrich. This Cy3 dye contains derivatized CyDye™, with only one reactive group on each dye molecule, ensuring precise labeling of amine groups in proteins (source: https://www.sigmaaldrich.com/US/en/product/sigma/gepa23001). Zeba spin desalting columns (7K MWCO) were purchased from Thermo Fisher Scientific.

The probing solutions and the 7-well and 14-well lectin chips, respectively coated with 45 and 9 distinct lectin proteins, were obtained from GlycoTechnica (Japan). Recombinant exoglycosidases β-N-acetylglucosaminidase S, α1–3,6 Galactosidase, and α2–3,6,8 Neuraminidase, as well as recombinant endoglycosidase Endo H, all expressed in *E. coli*, were purchased from New England BioLabs (NEB). Glycosyltransferases β(1,4)-galactosyltransferase and α2,3-sialyltransferase were obtained from Agilent. All enzymes were supplied with respective reaction buffers and substrates (for glycosyltransferases only).

### Lectin microarray analysis

We followed the experimental procedures developed by the LecChip maker, GlycoTechnica (Japan), which involve overnight incubation to ensure the detection of all binding signals, including weak binding signals. These same protocols have been used in our previously published studies.^16^ Protein samples of interest were prepared at 50 μg/mL in Tris-buffered saline (TBS). Aliquots 20 μL, containing 1 μg of protein, were mixed with 100 μg of Cy3 mono-reactive dye and incubated in the dark at 25°C for 1–2 hours on an Eppendorf thermomixer with a mixing speed of 300 rpm. To remove the unbound Cy3 dye, Zeba spin desalting columns (7K MWCO) were used. The Cy3-labeled protein samples were serially diluted to 125 ng/mL in the probing solution. Subsequently, aliquots of 40 μL or 100 μL was applied to the wells of 14-well or 7-well lectin chips, respectively. The lectin chips were kept overnight in the dark at room temperature on an orbital shaker. Afterward the fluorescence intensity at each lectin-coated spot was scanned using an evanescent-field fluorescence scanner called GlycoStation Reader 2300 (GlycoTechnica, Japan), without any washing steps. The obtained fluorescence intensities were analyzed using the GlycoStation Tool SignalCapture 3.0 software and GlycoStation ToolsPro3.0 software.

### In vitro glycoengineering of IgG1 mAbs

The terminal glycans of IgG1 mAbs were modified through in vitro enzymatic glycoengineering reactions at 37°C, using a combination of glycosidases (to remove glycans) and glycosyltransferases (to add glycans). Briefly, the following steps were taken: 1) To remove the terminal non-reducing β-N-acetylglucosamines, 10 μg of benralizumab or obinutuzumab was diluted to 0.2 mg/mL in GlycoBuffer-1 (supplied at 10× concentration consisting of 0.5 M sodium acetate and 50 mM CaCl_2_ at pH 5.5). It was then mixed with 40 units of β-N-acetylglucosaminidase S and incubated for 3.5 hours; 2) To remove the chitobiose core of high mannose, 10 μg of siltuximab was diluted to 0.2 mg/mL in GlycoBuffer-3 (supplied at 10× concentration as 500 mM sodium acetate, pH 6.0). It was mixed with 2,500 units of Endo H and then incubated overnight; 3) To remove the terminal α2–3 and α2–6 linked sialic acids (desialylation), 10 μg of ramucirumab was diluted to 0.2 mg/mL in GlycoBuffer-1. It was mixed with 50 units of α2–3,6,8 neuraminidase and then incubated for 6 hours; 4) To remove terminal α1–3 and/or α1–6 linked α-galactoses, 10 μg of ramucirumab was diluted to 0.2 mg/mL in GlycoBuffer-1. It was mixed with BSA (supplied as 100× concentration) and 20 units of α1–3,6 galactosidase, then incubated for 6 hours; 5) To engineer a terminal β(1,4)-galactose onto GlcNAcβ1-2Man (β-galactosylation), 100 μg of NISTmAb or obinutuzumab was diluted to 2 mg/mL in Reaction Buffer (supplied at 5× concentration consisting of 50 mM MnCl2 and 500 mM MES at pH 6.5). It was mixed with 3 μg of β(1,4)-galactosyltransferase and 120 μg of substrate uridine-5’-diphosphogalactose disodium salt (UDP-Gal), then incubated for 6 hours; 6) To further add terminal α2,3-linked sialic acid NANA to Galβ(1–4)GlcNAc (+ galactosylation + α2,3-NANA), 20 μg of the above galactosylated NISTmAb was diluted to 0.5 mg/mL in Reaction Buffer (supplied at 5× concentration as 1 M MES, pH 6.5). It was mixed with 2 μg of α2,3-sialyltransferase and 10 μg of substrate cytidine 5’-monophospho-N-acetylneuraminic acid disodium salt (CMP-NANA), then incubated overnight. The resulting glycoengineered protein samples from these reactions were stored at −20°C until further analyses.

### LC-MS analysis

Liquid chromatography coupled with mass spectrometry (LC-MS) analyses were conducted using an Agilent 1260 HPLC-Chip nano-electrospray-ionization 6520 Q-TOF MS system. The solvents used were A and B, both containing 0.1% formic acid, but in water and 95% acetonitrile, respectively. To ensure accurate mass measurement, mass correction was enabled using internal reference ions of known masses of 299.2945 and 1221.9906 Dalton. For intact protein mass measurement, an Agilent 43 mm 300 Å C8 chip with a 40 nL trap column (G4240–63001) were employed. All IgG1 mAb samples were first reduced in 50 mM dithiothreitol for 30 minutes at 37°C, followed by the addition of 0.1% formic acid (v/v). After centrifugation at 18,000×g for 2 minutes, the supernatant containing 40.9 ng/μL IgG1 (~0.5 μM heavy chain) was transferred to an HPLC vial. Subsequently, 2 μL (~1 pmol heavy chain) was injected onto the trap column in the C8 chip at a flow rate of 2.5 μL/min, using 100% solvent A. The elution was carried out at 0.5 μL/min with a linear gradient from 10% to 100% solvent B over 18 min, followed by a 4-minutes hold. To prevent any potential carryover issues between two sample runs, a blank run of solvent A was conducted. The Q-TOF VCap, fragmentor, and skimmer settings were set at 1,890 eV, 225 eV, and 65 eV, respectively. The HPLC-Chip gas temperature and drying gas flow rate were maintained at 350°C and 9 L/min, respectively. Data analysis was performed using Agilent MassHunter (version B.05.00) Qualitative Analysis software, and deconvoluted was carried out using Agilent MassHunter Bioconfirm software.

## Supplementary Material

Supplemental Materials R2.docx
