# Supplementary material for: A tailored lectin microarray for rapid glycan profiling of therapeutic monoclonal antibodies
Source: MAbs. 2024 Jan 22;16(1):2304268. doi: 10.1080/19420862.2024.2304268 (PMC10807468; doi:10.1080/19420862.2024.2304268)
Supplement: Supplemental Materials R2.docx [file KMAB_A_2304268_SM3875.docx]

**Supplemental Materials**

Supplemental Table 1. Reported glycan selectivity of the 45 natural lectins used in this study*

| No. | Lectin (origin) | Glycan selectivity | No. | Lectin (origin) | Glycan selectivity |
| --- | --- | --- | --- | --- | --- |
| 1 | LTL (*Lotus tetragonolobus*) | Fucα1-3(Galβ1-4)GlcNAc (Lewis x), Fucα1-2Galβ1-4GlcNAc (H-type 2) | 24 | TJA-II (*Tanthes japonica*) | Fucα1-2Galβ1-> or GalNAcβ1-> groups at their non-reducing terminals |
| 2 | PSA (*Pisum sativum*) | Fucα1-6GlcNAc (Core Fuc) , α-Man | 25 | EEL (*Euonymus europaeus*) | Galα1-3Galβ1-4GlcNAc, Fucα1-2Galβ1-3GlcNAc (H antigen) |
| 3 | LCA (*Lens culinaris*) | Fucα1-6GlcNAc (Core Fuc), α-Man | 26 | ABA (fungus, *Agaricus bisporus*) | Galβ1-3GalNAc, GlcNAc |
| 4 | UEA-I (*Ulex europaeus*) | Fucα1-2Galβ1-4GlcNAc (H-type 2) | 27 | LEL (tomato, *Lycopersicon esculentum*) | (GlcNAcβ1-4)n (Chitin), (Galβ1-4GlcNAc)n (polylactosamine) |
| 5 | AOL (*Aspergillus oryzae*) | Fucα1-6GlcNAc (Core Fuc), Fucα1-2Galβ1-4GlcNAc (H-type 2) | 28 | STL (potato, *Solanum tuberosum*) | (GlcNAcβ1-4)n (Chitin), oligosaccharide containing GlcNAc and MurNAc |
| 6 | AAL (*Aleuria aurantia*) | Fucα1-3(Galβ1-4)GlcNAc (Lewis x), Fucα1-6GlcNAc (Core Fuc) | 29 | UDA (*Urtica dioica*) | GlcNAcβ1-4GlcNAc (Chitin), High-Mannose (3 to High, up with increasing the number of Man) |
| 7 | MAL_I (*Maackia amurensis*) | Siaα2-3Galβ1-4GlcNAc | 30 | PWM (pokeweed, *Phytolacca Americana*) | (GlcNAcβ1-4)n (Chitin) |
| 8 | SNA (*Sambucus nigra*) | Siaα2-6Gal/GalNAc | 31 | Jacalin (*Artocarpus integrifolia*) | GlcNAcβ1-3GalNAc (Core3), Siaα2-3Galβ1-3GalNAc (sialyl T), Galβ1-3GalNAc (T-antigen), α-GalNAc (Tn-antigen) |
| 9 | SSA (*Sambucus sieboldiana*) | Siaα2-6Gal/GalNAc | 32 | PNA (peanut, *Arachis hypogaea*) | Galβ1-3GalNAc |
| 10 | TJA-I (*Trichosanthes japonica*) | Siaα2-6Gal/GalNAc, HSO3(-) -6Galβ1-4GlcNAc | 33 | WFA (*Wisteria floribunda*) | GalNAcβ1-4GlcNAc (LacdiNAc), Galβ1-3(-6)GalNAc |
| 11 | PHAL (*Phaseolus vulgaris*) | tri/tetra-antennary complex-type N-glycan | 34 | ACA (*Amaranthus caudatus*) | Galβ1-3GalNAc (T-antigen), Siaα2-3Galβ1-3GalNAc (sialyl T) |
| 12 | ECA (*Erythrina cristagalli*) | Galβ1-4GlcNAc (up with increasing the number of terminal Gal), no affinity for fully sialylated N-type, fully agalactosylated N-type | 35 | MPA (*Maclura pomifera*) | α-GalNAc (Tn-antigen), Galβ1-3GalNAc (T-antigen), |
| 13 | RCA120 (*Ricinus communis*) | Galβ1-4GlcNAc (up with increasing the number of terminal Gal), Galβ1-3Gal (weak), no affinity for agalactosylated N-type | 36 | HPA (snail, *Helix pomatia*) | α-GalNAc |
| 14 | PHAE (*Phaseolus vulgaris*) | bi-antennary complex-type N-glycan with outer Gal and bisecting GlcNAc, no affinity for fully sialylated N-type | 37 | VVA (*Vicia villosa*) | GalNAcβ1-4Gal, GalNAcβ1-3Gal, α-GalNAc |
| 15 | DSA (*Datura stramonium*) | (GlcNAcβ1-4)n, tri/tetra-antennary N-glycan | 38 | DBA (*Dolichos biflorus*) | Blood group A, GalNAcα1-3GalNAc, GalNAcβ1-4(Siaα2-3)Galβ1-4Glc (GM2) |
| 16 | GSL-II (*Griffonia simplicifolia*) | agalactosylated tri/tetra antennary glycans, GlcNAc, no affinity for fully galactosylated or sialylated N-type | 39 | SBA (soybean, *Dolichos biflorus*) | α- or β-linked GalNAc, Galα1-4Gal-Glc |
| 17 | NPA (*Narcissus pseudonarcissus*) | High-Mannose including Manα1-6Man | 40 | Calsepa (*Calystegia sepium*) | Galactosylated bianntenary N-type with bisecting GlcNAc (galacto > agalacto, down with Core Fuc), High-Mannose (Man2-6) |
| 18 | ConA (*Canavalia ensiformis*) | High-Mannose including Manα1-6(Manα1-3)Man | 41 | PTL-I (*Psophocarpus tetragonolobus*) | α-GalNAc, Galα1-3(Fucα1-2)Gal (B-antigen) |
| 19 | GNA (*Galanthus nivalis*) | High-Mannose including Manα1-3Man | 42 | MAH (*Maackia amurensis*) | Siaα2-3Galβ1-3(Siaα2-6)GalNAc (disialyl-T) |
| 20 | HHL (*Hippeastrum hybrid*) | High-Mannose including Manα1-3Man or Manα1-6Man | 43 | WGA (wheat germ, *Triticum aestivum*) | (GlcNAcβ1-4)n (Chitin), Hybrid type N-glycan, Sia |
| 21 | ACG (mushroom, *Agrocybe cylindracea*) | Galβ1-3Gal, Siaα2-3Galβ1-4GlcNAc | 44 | GSL-I A4 (*Griffonia simplicifolia*) | α-Gal, α-GalNAc |
| 22 | TxLCI (*Tulipa gesneriana*) | Manα1-3(Manα1-6)Man, bi/tri-antennary complex-type N-glycan, GalNAc | 45 | GSL-I B4 (*Griffonia simplicifolia*) | α-Gal, α-GalNAc |
| 23 | BPL (*Bauhinia purpurea*) | Galβ1-3GalNAc (up with Lewis x, down with Core Fuc), GalNAc |  |  |  |

** Refer to Lectin Frontier DataBase (LfDB)(https://acgg.asia/lfdb2/).*

Supplemental Table 2. Glycan selectivity of the 29 recombinant lectins used in this study*

| **No.** | **Lectin**** | **Glycan selectivity** | **No.** | **Lectin**** | **Glycan selectivity** |
| --- | --- | --- | --- | --- | --- |
| 1 | rBC2LCN | Fucose | 16 | rMan3 | High Mannose Type I |
| 2 | rPhoSL | Core fucose | 17 | rMan4 | High Mannose Type II |
| 3 | rCfuc | Core fucose | 18 | rMan5 | High Mannose Type III |
| 4 | rCGL2 | Gal/GalNAc-Complex | 19 | rMan6 | High Mannose Type IV |
| 5 | rCNL | Gal/GalNAc-Complex | 20 | rMan7 | High Mannose |
| 6 | rDiscoidin I | Gal/GalNAc-Complex | 21 | rMan8 | High Mannose |
| 7 | rDiscoidin II | Gal/GalNAc-Complex | 22 | rMan9 | High Mannose |
| 8 | rGal9 | Gal/GalNAc-Complex | 23 | rOGH1 | O-glycan |
| 9 | rLSL-N | Gal/GalNAc-Complex | 24 | rOGH2 | O-glycan |
| 10 | rMOA | (α-Gal)Gal/GalNAc-Complex | 25 | rOGH3 | (GalNAc) O-glycan |
| 11 | rF17AG | Glc/GlcNAc-Complex | 26 | rOTH1 | Unknown |
| 12 | rMalectin | Glc/GlcNAc-Complex | 27 | rOTH2 | Unknown |
| 13 | rPSL1a | Sialic Acids | 28 | rOTH3 | Unknown |
| 14 | rMan1 | High Mannose Type I | 29 | rCSL | Rhamnose |
| 15 | rMan2 | High Mannose Type I |  |  |  |

** Provided by the LecChip manufacturer GlycoTechnica.*

*** Company codes for recombinant lectins.*

Supplemental Table 3. List of lectins with glycan-independent interaction with nonglycosylated IgG1 mAb atezolizumab, as observed in supplemental Figure 1.

| No. | 1 | 2 | 3 | 4 | 5 | 6 | 7 | 8 | 9 |
| --- | --- | --- | --- | --- | --- | --- | --- | --- | --- |
| Lectin | LCA | DSA | ConA | LEL | STL | UDA | ACA | rDiscoidin I | rDiscoidin II |
| No. | 10 | 11 | 12 | 13 | 14 | 15 | 16 | 17 |  |
| Lectin | rGal9 | F17AG | Man3 | rMan6 | rOGH1 | rOGH3 | rOTH1 | rCSL |  |

Supplemental Figure 1. Lectin-based glycan profiles of non-glycosylated protein filgrastim, N-glycosylated IgG1 reference material NISTmAb, and non-glycosylated IgG1 atezolizumab. Glycan profiles were obtained by using (a) the 45 natural lectins listed in supplemental Table-1 and (b) the 29 recombinant lectins listed in supplemental Table-2. Any observed green and red signals indicate glycan-independent IgG1—lectin and protein—lectin interaction, respectively. Note that the last two groups of signals represent position marker and background (BG) signals on the LecChip. The chips were scanned using older generation scanner GlycoStation Reader 1200.

Supplemental Figure 2. Reported PHAE interactions with bisecting and non-bisecting glycans. The binding affinities of PHAE for pyridylaminated glycans, which correspond to and are reported as the V-V0 values (a), were obtained from the Lectin Frontier DataBase (<https://acgg.asia/lfdb2/>). The top three PHAE-binding glycans are bisecting glycans bG2, bG2F, and bG1 (b). However, none of these glycans was abundant in obinutuzumab (see Figure 3e). Instead, the two most abundant glycans detected in obinutuzumab, bG0 and bG0F, have similar binding affinities for PHAE as several non-bisecting glycans commonly found on therapeutic mAbs, including the top three abundant glycans G0F, G1F and G2F (c). These interactions explain the observed PHAE-binding signal in many mAb samples (Figures 3 & 4) although they contained trace amounts of undetectable levels of bisecting glycans.

Supplemental Figure 3. Assessment of glycosylation across various manufacturing batches. N-glycan profiles in three batches of infliximab drug product were compared by LecChip-IgG-mAb microarray. Shown are the glycan profiles derived from 1-second exposures. The error bar represents standard deviation (n=3) of three independent experiments. The results indicate comparable glycan profiles across three manufacturing batches.
